# A Minor Haplotype Variant Determines the Pathogenicity of the p.Ile279Thr Substitution in the Primary Hyperoxaluria Type 1 Gene, *AGXT*

**DOI:** 10.1002/jimd.70052

**Published:** 2025-06-11

**Authors:** Luana Ruta, Andrea G. Cogal, Gioena Pampalone, David J. Sas, John C. Lieske, Gill Rumsby, Barbara Cellini, Peter C. Harris

**Affiliations:** ^1^ Department of Medicine and Surgery University of Perugia Perugia Italy; ^2^ Division of Nephrology and Hypertension Mayo Clinic Rochester Minnesota USA; ^3^ Division of Pediatric Nephrology and Hypertension Mayo Clinic Rochester Minnesota USA; ^4^ Department of Laboratory Medicine and Pathology Mayo Clinic Rochester Minnesota USA; ^5^ University College London Hospitals London UK; ^6^ Department of Biochemistry and Molecular Biology Mayo Clinic Rochester Minnesota USA

**Keywords:** primary hyperoxaluria: polymorphism, pyridoxal phosphate: pathogenic varian, rare disease

## Abstract

Human alanine:glyoxylate aminotransferase (AGT) is a liver peroxisomal enzyme that metabolizes glyoxylate, the oxalate precursor, to glycine. AGT deficiency, due to recessive pathogenic changes in the *AGXT* gene, results in calcium oxalate accumulation and kidney stones, a condition known as primary hyperoxaluria type 1 (PH1). Most missense variants lead to PH1 by causing AGT misfolding, but their effects manifest differently based on the presence of two polymorphic variants, p.Pro11Leu (p.P11L) and p.Ile340Met (p.I340M), which are usually present in linkage disequilibrium and generate the minor haplotype. While the p.P11L substitution reduces AGT global stability and worsens the effects of pathogenic changes, the p.I340M exerts a stabilizing effect whose role on PH1 pathogenesis has never been elucidated. The p.Ile279Thr (p.I279T) variant is frequent in healthy populations (0.29%), mainly on the major allele, but we present data from six PH1 families (eight patients) suggesting p.I279T as a PH1 pathogenic allele. Interestingly, in these families, p.Thr279 is always associated with p.Leu11 and p.Ile340, thus with a split *AGXT* haplotype. Analysis of the effects of the p.I279T mutation by in silico predictions, biochemical analyses on purified proteins, and expression in two cellular models of disease (*AGXT1*‐KO HepG2 and CHO cells) shows that it causes a folding defect that is exacerbated by p.P11L but mitigated by p.I340M, thus explaining why p.Thr279 is pathogenic just on the p.Leu11–p.Ile340 haplotype. These data indicate that genetic screenings for PH1 should document the *AGXT* haplotype, including its components, to obtain an accurate diagnosis and possible prognostic information.

## 1 Introduction

Human liver peroxisomal alanine‐glyoxylate transaminase (AGT) catalyzes the transamination of L‐alanine and glyoxylate to pyruvate and glycine, respectively, an irreversible reaction under physiological conditions, using pyridoxal 5′‐phosphate (PLP) as a coenzyme [1]. In humans, AGT is encoded by *AGXT*, and biallelic pathogenic variants are the major cause of primary hyperoxaluria type 1 (PH1; ~80% of primary hyperoxaluria cases). Reduced activity of AGT results in glyoxylate accumulation, which leads to overproduction of oxalate and glycolate [2]. Insoluble calcium oxalate crystals aggregate, leading to nephrolithiasis, nephrocalcinosis, and often kidney failure.

*AGXT* is expressed on either a major (Ma; ~82%) or a minor (Mi; ~18%) haplotype, the latter characterized by a 74 bp‐duplication in intron 1 and by two base‐pair changes (c.32C>T and c.1020A>G) leading to the p.Pro11Leu (p.P11L) and p.Ile340Met (p.I340M) amino acid substitutions, respectively [3]. The molecular and cellular effects of these missense changes indicate that alone the minor haplotype is not pathogenic. However, AGT‐Mi has reduced thermodynamic and kinetic stability, which translates to an increased susceptibility to unfolding and aggregation both in the purified form and in cellular systems, where AGT‐Mi also shows a reduced half‐life [4, 5, 6, 7, 8]. Notably, the p.P11L variant creates a weak mitochondrial targeting sequence that is not functional on AGT‐Mi because the latter folds and dimerizes quickly enough to avoid mitochondrial import [3]. However, when a PH1‐associated pathogenic change is inherited on AGT‐Mi, a synergism can occur between the pathogenic and polymorphic changes, so that a severe folding defect and/or aberrant mitochondrial mistargeting arise leading to AGT deficiency and PH1 [5, 9, 10, 11]. Synergism explored at the biochemical level indicates that the pathogenicity or severity of some variants is influenced by the haplotype, such as for the most common PH1 pathogenic variant, p.Gly170Arg (p.G170R) that is found on the minor haplotype [12, 13, 14].

Interestingly, the in‐depth characterization of the two substitutions typical of the minor allele has shown that the substitution p.P11L is mainly responsible for the increased overall dynamics and reduced stability of AGT‐Mi [8, 15, 16], as recently confirmed [17, 18]. Notably, the p.I340M change exerts a stabilizing role that partly compensates for the effects of the p.P11L substitution [15, 17], but how it influences the molecular phenotype of pathogenic mutations is unknown.

AGT is dimeric and belongs to the Fold Type I family of PLP enzymes. p.Ile279 (p.I279) is located in an external region that packs against the N‐terminus of the neighboring subunit where p.Pro11 (p.P11) is located. The c.836 T>C; p.Ile279Thr (p.I279T) substitution is quite common in the general population (0.29% in gnomAD v4.1.0), usually on the major haplotype, and it does not change the specific activity of AGT‐Ma when expressed in bacteria [19]. However, there is anecdotal evidence that p.I279T may sometimes be pathogenic, being the second possible variant in compound heterozygous cases, and in some of these cases shown to present with the p.Leu11 (p.L11) allele [19, 20, 21], prompting a ClinVar entry (CodeVCV002681193). However, the consequences of the *AGXT* haplotype on this variant have not been studied. Here, we dissect the haplotype in six PH families with p.I279T and characterize in detail the biochemical effects of this substitution on different haplotypes.

## 2 Materials and Methods

### 2.1 Materials

PLP, L‐alanine, sodium glyoxylate, rabbit muscle lactate dehydrogenase (LDH), isopropyl‐β‐D‐1‐thiogalactopyranoside (IPTG), and imidazole were purchased from Merck Life Science srl (St. Louis, MO, USA). Oligonucleotides for site‐directed mutagenesis were purchased from Bio‐Fab Research srl (Rome, Italy) Sigma. Protease Inhibitor Cocktail EDTA‐free and Protease Inhibitor Cocktail Complete Mini were purchased from Roche. All other chemicals were of the highest purity available.

### 2.2 Methods

#### 2.2.1 Genetic and Clinical Studies

This study was reviewed and approved by the Institutional Review Board of the Mayo Clinic Rochester, MN (Title: Characterization of Monogenic Kidney Stone Diseases IRB#: 17–005513, initially approved September 8, 2017, last modified April 28, 2025). All procedures were conducted in accordance with the ethical standards of the institution and the 1964 Helsinki Declaration and its later amendments. Written informed consent was obtained from all participants prior to enrollment. Families with *AGXT* p.I279T identified as a possible pathogenic change (Table 1) were typed for the *AGXT* haplotype markers by Sanger and/or next‐generation sequencing, as described [24]. Clinical and biochemical data on the affected individuals were extracted from available clinical records.

**TABLE 1:** Haplotype and details of the families with possibly clinically significant *AGXT,* p.Ile279Thr.

			p.Ile279Thr haplotype									
Ped #: Ind #	Pub	Sex	p.P11L	p.I340M	2nd Path Var	HyOx, age	NL, age	NC, age	Oxal, age	AGT Func	KF, age	Comments
1: 2–1	182–01 [22]	F	p.L11^a^	p.I340^a^	c.972delG; p.Ala325fs	POx = **60**, 18 y	M, 3 y	Y, 3 y	Y, 3 y	NA	Y, < 18 y	Figure 1A; KT
1: 2–2	182–01 [22]	M	p.L11^a^	p.I340^a^	c.972delG; p.Ala325fs	POx 62.5, 16 y	M, 0 y	Y, 0 y	Y, 16 y	NA	Y, 2 y	Figure 1A; KLT
2	NP	F	p.L11^a^	p.I340^b^	c.508G>A; p.Gly170Arg	UOx = 1.9, 35y	M, 17 y	Y, 17 y	Y, 40 y	NA	Y, 41 y	Figure 1B; KLT
3: 2–1	P31 [21]	M	p.L11^a^	p.I340^b^	c.508G>A; p.Gly170Arg	Y,? y	M, 13 y	Y, 13 y	Y	NA	Y, 42 y	Figure 1C; KLT
3: 2–2	P31 [21]	F	p.L11^a^	p.I340^b^	c.508G>A; p.Gly170Arg	1.5, 35 y	Y, 15 y	N	N	NA	N, 50 y	Figure 1C
4	NP	M	p.L11^a^	p.I340^a^	^1^c.836 T>C; p.Ile279Thr	UOx = **3**, 56y Ugly = **1.4**, 56y	17B,? y	NA	NA	10%	N, 56 y	
5	F7 [23]	F	p.L11^a^	ND	c.560C>T; p.Ser187Phe	Y,? y	M, 12 y	Y, < 12 y	NA	7%	Y, 15 y	
6	NP	F	p.L11^a^	p.I340^c^	c.121G>A; p.Gly41Arg	POx = **157**	R, ~15 y	NA	NA	9%	Y, < 57 y	

*Note:* Ped #, pedigree number; Ind #, individual number. Pub, previous identifier and publication; NP, not published. Sex: F, female; M, male. How phase determined: a, homozygous; b, segregation; c, inferred from haplotype of 2nd allele; ND, not determined. HyOx, Hyperoxaluria; UOx, Urine Oxalate, mmol/24 h (normal range *0.11–0.46*); UGly, Urine glycolate, mmol/24 h (normal range *0.14–0.62*); POx, Plasma oxalate, umol/L (normal range < 10); Elevated values are emboldened. NL, nephrolithiasis: Y, yes, age; B, bilateral; M, multiple; *R*, recurrent. NC, nephrocalcinosis: Y, yes, age; *N*, no, age; ?, unknown age; NA, information not available. Oxal, Oxalosis; Y, yes, age; NA, information not available. AGT Func, percent AGT function determined from liver biopsy: NA, information not available. KF, kidney failure: Y, yes, age; *N*, no, age. ?, unknown age. Comments: Figure 1, figure showing the pedigree; KLT, kidney/liver transplant; KT, kidney transplant.

^1^ Homozygous for p.Ile279Thr.

#### 2.2.2 In Silico Analyses

Starting from the crystal structures of AGT‐Ma (PDB ID: 5F9S) and AGT‐Mi (PDB ID: 7NS7) we predicted the structural effects of the p.I279T and p.P11L variants using (i) *AlphaMissense*, which scores missense mutations based on the sequence conservation; (ii) *DynaMut*, *DDGun, and DDMut*, which predict the ΔΔG of unfolding upon single and/or multiple site variants; and (iii) *MutPred2*, a machine learning‐based method that gives probabilistic quantitative predictions about the general pathogenicity of amino acid substitutions and a list of molecular alterations potentially affecting the phenotype based on the protein sequence [25].

To rationalize the local effects of p.I279T on the AGT structure, we performed molecular modeling analyses through UCSF Chimera (alpha version 1.17). Briefly, the dimeric structures of AGT‐Ma and AGT‐Mi (5F9S and 7NS7) were used as templates to predict the structures of the variants using the Structure Editing tool, and the possible steric clashes/contacts with residues on a 5 Å range using the Structure Analyses tool.

#### 2.2.3 Site‐Directed Mutagenesis

The pTrcHis2A‐AGT‐Ma, pTrcHis2A‐AGT‐Mi, pcDNA3.1‐AGT‐Ma, and pcDNA3.1AGT‐Mi vectors for the expression of AGT‐Ma and AGT‐Mi in bacterial and mammalian cells were already available [1]. Each variant was introduced by site‐directed mutagenesis using the Phusion Hot Start II High‐Fidelity PCR Master Mix (Thermo Fisher Scientific, Waltham, MA, USA). We used the mutagenic primers 5’ GCTGCTGGTGACCCCCCTCAAGGCCCTGCTCAAGC 3′ and its complement to introduce p.P11L on AGT‐Ma (AGT‐L11). We then used the mutagenic primers 5’AGAGAGAGCCTGGCCCTCACTGCGGAACAG 3′ and its complement to introduce p.I279T onto three different backgrounds (T279‐Ma, T279‐Mi and T279‐L11). A complete list of the haplotypes is reported in Table 2. The thermal profile, digestion of PCR products with DpnI (Thermo Fisher Scientific, Waltham, MA, USA), and transformation of DH5α *E. coli* cells were performed as previously described [1]. Plasmids were extracted using the GenElute miniprep kit (Merck Life Science S.r.l, St. Louis, MO, USA). The correct introduction of each variant was verified by Sanger sequencing (Bio‐Fab Research S.r.l, Rome, Italy) followed by sequence analysis using Chromas (V2.6.6, Technelysium Pty Ltd., South Brisbane, QLD, Australia).

**TABLE 2:** Schematic representation of the enzymatic species under study and their haplotype.

Species	Haplotype		
AGT‐Ma	P11	I279	I340
AGT‐Mi	L11	I279	M340
AGT‐L11	L11	I279	I340
T279‐Ma	P11	T279	I340
T279‐Mi	L11	T279	M340
T279‐L11	L11	T279	I340

#### 2.2.4 Protein Expression and Purification

AGT‐Ma, AGT‐Mi, AGT‐L11, and the p.I279T mutant proteins (T279‐Ma, T279‐Mi, T279‐L11) in their His‐tagged form were expressed in *E. coli* BL21(DE3) cells grown in Luria broth medium at 37°C. Expression was induced with 0.1 mM IPTG for 15 h at 30°C. Cells were harvested and resuspended in 20 mM NaH_2_PO_4_, 500 mM NaCl, 20 mM imidazole (buffer A) plus 100 μM PLP pH 7.4, and protease inhibitor cocktail cOmplete ULTRA tablets. Lysozyme was added to a concentration of 0.2 mg/mL, and the culture was incubated for 15 min at room temperature. After freezing at −80°C, thawed lysate was centrifuged at 18000 × *g* for 30 min at 4°C. The supernatant was loaded on a HisPrep FF 16/10 (GE Healthcare, United States) column previously equilibrated in buffer A. The protein was eluted using a linear gradient of 0%–100% 20 mM NaH_2_PO_4_, 500 mM NaCl, 500 mM imidazole, pH 7.4 in 100 mL, and the fractions containing AGT were pooled, reconstituted with 100 μM PLP, concentrated, and dialyzed in 100 mM potassium phosphate (KP) buffer, pH 7.4, using Amicon Ultra (Millipore, Germany). Protein concentration was determined using the molar extinction coefficient of 95 400 M^−1^ cm^−1^ at 280 nm [26]. The PLP content was determined by releasing the coenzyme in 0.1 M NaOH and using the molar extinction coefficient of 6600 M^−1^ cm^−1^ at 388 nm.

#### 2.2.5 Activity Assays

AGT transaminase activity was determined by a spectrophotometric assay based on the use of LDH as a coupled enzyme [27]. A typical mixture contained 0.1–0.2 μM purified proteins or 80 μg cellular lysate, 200 μM PLP, and 10 mM glyoxylate, in 100 mM KP pH 7.4, and the reaction was started by adding 0.5 M L‐alanine. The mixture was incubated for 10–20 min at 25°C, and the reaction was stopped by adding trichloroacetic acid (TCA) 10% (v/v). Pyruvate production was quantified in 370 mM Tris–HCl, pH 8.3 buffer in the presence of 360 μM NADH and 13 μg/mL LDH by monitoring the change in absorbance at 340 nm using the molar extinction coefficient of NADH (6220 M^−1^ cm^−1^). Kinetic parameters were measured by varying the L‐alanine concentration in the presence of a fixed concentration of glyoxylate and vice versa and fitting the data to a Michaelis–Menten equation.

#### 2.2.6 Spectrophotometric and Spectrofluorometric Analyses

Absorption spectra were registered using a Jasco V‐750 spectrophotometer (Jasco Europe S.r.l., Cremella, LC) at a protein concentration of 0.5–1 mg/mL. Fluorescence spectra were registered by an FP8200 Jasco spectrofluorometer using 5 nm bandwidths at a protein concentration of 0.1 mg/mL. Spectra of samples containing all components except AGT (blanks) were taken immediately prior to the measurements of samples containing enzyme. CD spectra were obtained with a Jasco J‐810 spectropolarimeter with a thermostatically controlled cell compartment at 25°C. For near‐UV and visible wavelengths, protein concentration was 1–2 mg/mL in the presence of 20 μM PLP in a cuvette with a 1 cm path length. For far‐UV CD measurements, protein concentration was 0.1 mg/mL, and the path length was 0.1 cm.

#### 2.2.7 Stability Studies

AGT‐Ma, AGT‐Mi, AGT‐L11, and the T279 mutants at 0.1–0.2 μM protein concentration were incubated at 37°C in 100 mM KP pH 7.4 in the presence of 200 μM PLP, and the time‐dependent changes in enzymatic activity were measured. To evaluate the transition midpoints of thermal denaturation, the enzymes (0.1 mg/mL) in 100 mM KP pH 7.4 were incubated with 1X SYPRO Orange Probe (5000X in DMSO, Thermo Fisher Scientific, Waltham, MA, USA) on a Jasco FP‐8200 spectrofluorometer with excitation and emission wavelengths set at 490 nm and 530 nm, respectively. Continuous temperature increases from 25°C to 100°C with a ramp increment of 2.1°C/min was applied, and fluorescence was registered every 0.5 s. A control with all mixture components but the protein was also included as a blank for each measurement. Turbidimetry experiments were carried out by measuring the time‐dependent changes in absorbance at 600 nm of 10 μM protein in the presence of 10 μM PLP in PBS 1X, in a final volume of 200 μL using a MultiSkan SkyHigh microplate (Thermo Fisher Scientific, Waltham, MA, USA) reader at 37°C. To test the susceptibility to proteolysis, 7.5 μg of each protein were incubated with proteinase K at a 100:1 (w/w) protein/protease ratio at 25°C in 100 mM KP, pH 7.4. At different time points (2, 5, 15, 30, and 50 min), aliquots were removed, denatured in sample buffer 4× (1 M Tris–HCl, pH 6.8, 50% glycerol, 10% sodium dodecyl sulfate, 1% bromophenol blue, and 4% β‐mercaptoethanol) boiled for 5 min, and loaded on a 12% SDS‐PAGE gel. The gel was stained with the Colloidal Blue Staining Kit (Thermo Fisher Scientific, Waltham, MA, USA), destained with water overnight, and finally, visualized using the iBright FL1500 Imaging System (Thermo Fisher Scientific, Waltham, MA, USA).

#### 2.2.8 Cell Culture, Cell Transfection, and Immunoblot Analysis

*AGXT1*‐KO HepG2 and CHO cells were maintained as previously described [28, 29]. *AGXT1*‐KO HepG2 cells were transiently transfected with a pcDNA3.1/V5‐TOPO vector encoding for AGT‐Ma, AGT‐Mi, AGT‐L11, and the T279 mutants using the GenJet reagent (SignaGen Laboratories, MD, USA). As a negative control, cells were transfected with an empty vector. After 24 h, cells were either collected using Sample Buffer 1× (1 M Tris–HCl, pH 6.8, 50% glycerol, and 10% sodium dodecyl sulfate) at 100°C to obtain the total lysate or lysed by freeze/thawing (five cycles) in PBS pH 7.2 supplemented with 100 μM PLP and the protease inhibitor cocktail (Complete Mini, Roche). Under the latter conditions, the whole‐cell extract was separated by centrifugation (13.200 × *g*, 10 min, 4°C) to obtain the soluble fraction. Protein concentration in cell lysates was determined by a Bradford assay in triplicate.

For immunoblot analyses, 5 μg of protein lysate was loaded on a 12% SDS‐PAGE gel, transferred to a nitrocellulose membrane, and checked by Ponceau staining. The membranes were then stained with anti‐*AGXT1* (1:1000) in 5% (w/v) milk in TTBS (50 mM Tris–HCl pH 7.5, 150 mM NaCl, and 0.1% Tween 20) overnight at 4°C, extensively washed in TTBS, and then incubated with an HRP‐conjugated anti‐rabbit (1:5000) as a secondary antibody. Protein gel images were acquired using an iBright FL1500 Imaging System (Thermo Fisher Scientific, Waltham, MA, USA). A *β*‐tubulin (1:5000) antibody was used as a loading control. Immunocomplexes were visualized by an enhanced chemiluminescence kit (ECL, Pierce Biotechnology, Rockford, IL). Densitometric analysis was performed using iBright Analysis Software version 5.3.0 (Thermo Fisher Scientific, Waltham, MA, USA).

#### 2.2.9 Statistical Analysis

Results are given as mean ± SEM. Experiments were performed at least in triplicate. Statistical analyses were performed using GraphPad Prism version 6.0 (GraphPad Software, San Diego, CA, USA).

## 3 Results

### 3.1 Clinical and Haplotype Analysis of *AGXT* , p.Ile279Thr Families

**FIGURE 1:**
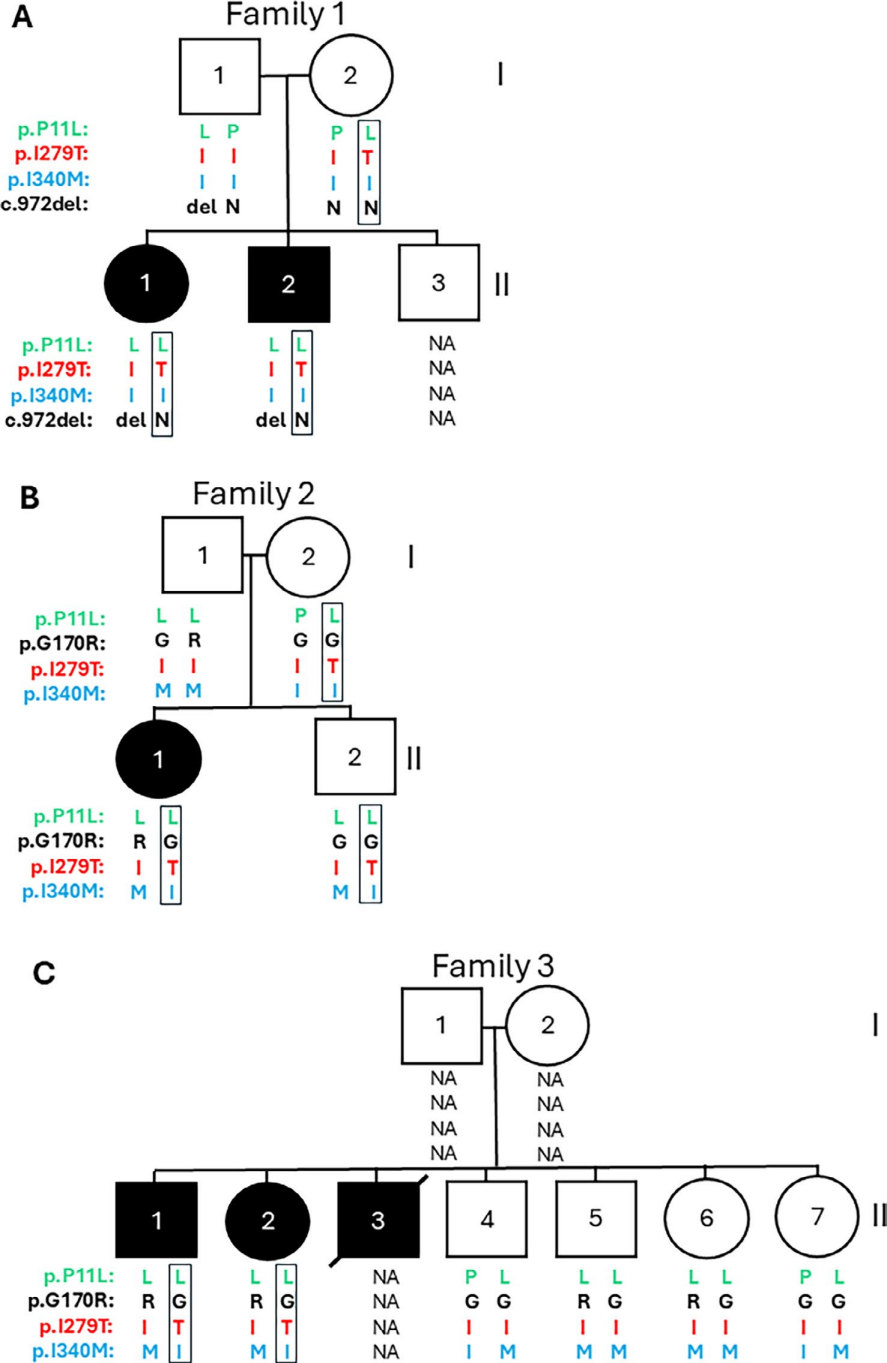
Pedigrees of three multiplex families (A–C) with *AGXT* p.I279T indicating haplotype data. The p.I279T variant is in red and the second pathogenic change in black, with the *AGXT* haplotype markers also shown: P.Pro11Leu (p.P11L; green) and p.Ile340Met (p.I340M; blue). The L11‐T279‐I340 haplotype that is always associated with the p.T279 allele is boxed. NA, data not available. Circles are females and squares males, with black‐filled shapes indicating affected individuals.

In six PH families (eight affected members) p.I279T was identified as possibly clinically significant because it was found in homozygosity (Family 4) or with a second known pathogenic change, without another likely significant pathogenic variant identified (Table 1). The affected individuals had clear evidence of PH1; three had biopsy‐proven reduction of AGT function, and six had kidney failure, from 2 to 57 years. Members from three of these families have previously been described [21, 22, 23]; families with two likely *AGXT* pathogenic changes plus p.I279T were excluded [21]. Sequence and segregation analysis of the pathogenic changes and the *AGXT* haplotype markers, p.P11L and p.I340M, showed conclusively in all individuals that p.T279 was on the AGT‐Mi‐p.L11 allele. However, further study proved or inferred that p.T279 was on a split p.L11/p.I340 haplotype (Table 1; Figure 1). Because of strong linkage disequilibrium, the AGT‐Ma and AGT‐Mi haplotypes are normally not split for these two markers, with p.T279 normally found on the intact AGT‐Ma haplotype, where it is functionally normal [21]. Given the genetic and clinical data and unusual haplotype, we explored if the haplotype variants influenced the functionality of AGT with the p.I279T substitution.

### 3.2 Bioinformatic Analyses of Predicted Effects of Ile279 Mutation

Employing AlphaMissense predictions, we found that the p.I279T substitution on AGT‐Ma gives a score of 0.22, which is compatible with it being benign, in line with the ClinVar classification (ClinVar code RCV000186256), previous functional data, and its frequency in healthy populations. Then we investigated in silico the effect of p.I279T on the overall stability of AGT with the different haplotypes (T279‐Ma; T279‐Mi; T279‐L11) (Table S1). According to DynaMut and MutPred2, the p.I279T substitution caused a decrease in protein stability on AGT‐Ma and in the presence of p.L11 alone, possibly associated with an increase in protein flexibility. Indeed, in both cases, DynaMut showed negative values for ΔΔG, mCSM, SDM, and DUET predictions, while MutPred2 showed a prediction score of 0.792 and 0.821 for T279‐Ma and T279‐L11, respectively, thus suggesting a high probability of pathogenicity. On the other hand, when p.T279 was present on the background of the minor allele, some of the DynaMut predicted parameters suggested a stabilizing effect and a decrease in flexibility, even if the MutPred2 score remained compatible with a pathogenic effect. Notably, no remarkable differences between the effects of the variant on the major and minor allele backgrounds are predicted by DDMut or DDGun servers.

Therefore, although in silico analyses mainly suggest a decreased AGT stability because of the p.I279T substitution, no clear conclusions about its effects and its possible synergism with substitutions typical of the *AGXT* minor haplotype can be drawn.

### 3.3 Molecular Effects of the p.I279T Variant on AGT in the Purified Form

To dissect the molecular effects of the p.I279T substitution in AGT, as well as its possible modulation by the concomitant presence of either of the two minor allele variants, we obtained T279‐Ma, T279‐Mi, and T279‐L11 in the purified form and compared their properties with the corresponding forms lacking the p.I279T substitution. The purity and the identity of the proteins were confirmed by SDS‐PAGE and western‐blot analyses, respectively. The purification yield of the mutant proteins was only slightly lower (50%–80%) as compared with that of AGT‐Ma, thus suggesting that the variant(s) do not significantly impact the expression in bacteria. This is in line with the far‐UV CD spectrum of the mutated species, which is comparable to that of AGT‐Ma, AGT‐Mi, and p.L11, thus revealing an analogous overall secondary structure composition (Figure S1A).

**FIGURE 2:**
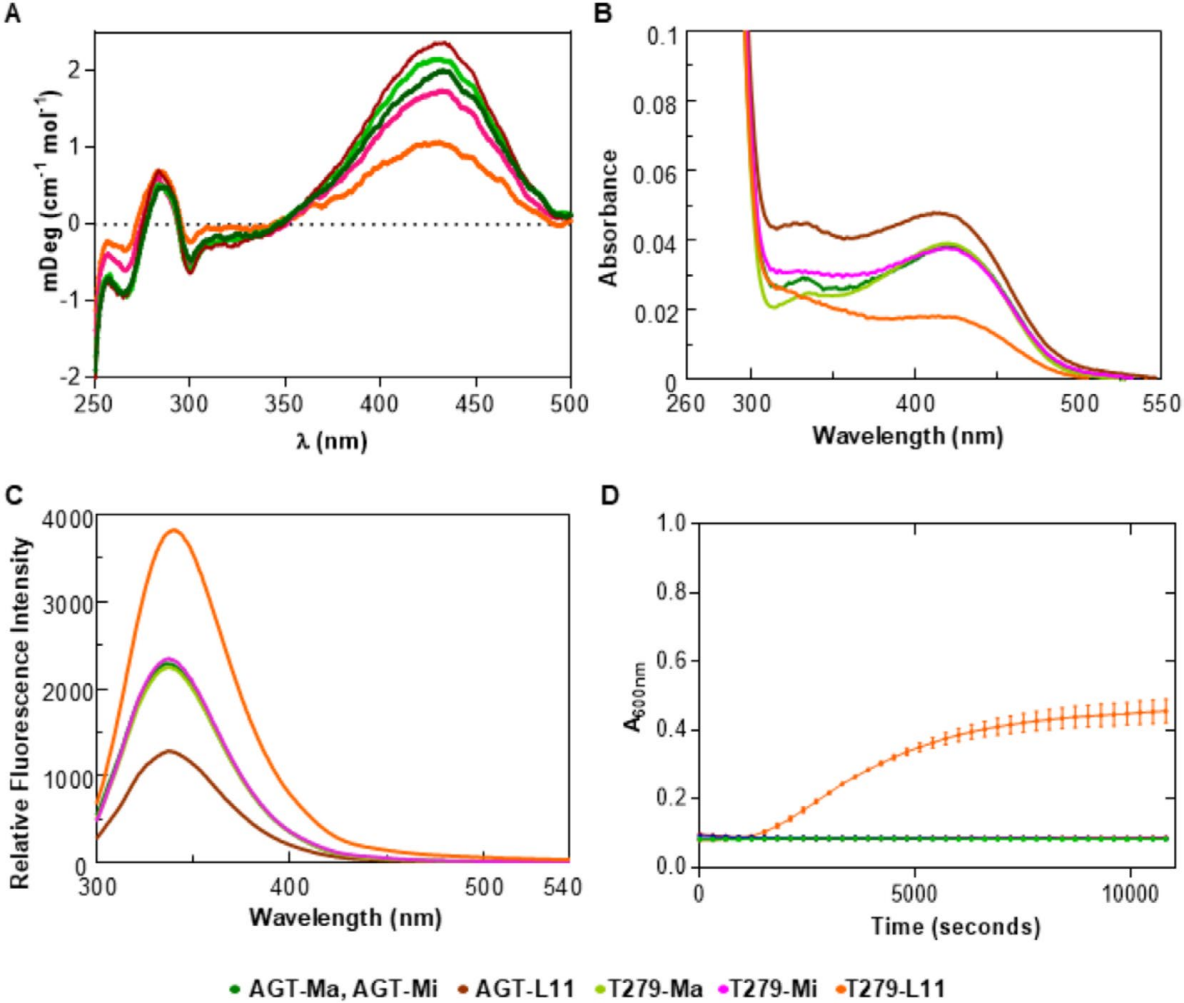
Molecular studies of AGT‐Ma, AGT‐Mi, AGT‐L11, and the p.T279 mutant proteins. (A) Near‐UV‐visible CD spectra in the presence of 20 μM exogenous PLP. (B) Absorbance spectra in the visible region. (C) Fluorescence emission spectra (excitation at 280 nm). All spectral data have been collected at 4 μM protein concentration in 100 mM KP pH 7.4 buffer. (D) Turbidimetry experiments on the selected mutants performed by monitoring time‐dependent changes in the absorbance at 600 nm. The experiments were performed by using 10 μM protein concentration at 37°C in 100 mM KP pH 7.4 in the presence of 20 μM exogenous PLP.

AGT‐Ma, AGT‐Mi, AGT‐L11 with or without p.T279 bind 2 mol of PLP per dimer. The CD and absorbance spectra in the visible region show the typical bands due to the coenzyme PLP bound at the active site, with an absorbance maximum centered at 423 nm and a shoulder at approximately 340 nm, attributable to the ketoenamine and enolimine tautomers of the internal aldimine, respectively (Figure 2A,B) [1]. The Abs_280_/Abs_423_ ratio is similar for AGT‐Ma, AGT‐Mi, and p.L11, while it slightly increases in T279‐Ma, T279‐Mi, and is approximately 2.5‐fold higher for T279‐L11. The band of the ketoenamine (Figure 2B) allowed calculation of the optical activity (expressed as millidegrees/absorbance at 410–430 nm), which was 97 mdeg/Abs_420_ nm for AGT‐Ma and AGT‐Mi, 122 mdeg/Abs_420_ nm for p.L11, 102 mdeg/Abs_420_ nm for T279‐Ma, 84 mdeg/Abs_420_ nm for T279‐Mi, and 41 mdeg/Abs_420_ nm for T279‐L11. The lowest optical activity observed for T279‐L11 indicates that the concomitant presence of p.L11 and p.T279 could increase the flexibility of the microenvironment of the internal aldimine. On the other hand, p.L11 alone displays an increased optical activity, thus suggesting that the single variant at the N‐terminus could increase the asymmetry of the active site. Upon excitation at 280 nm, a characteristic emission maximum at 337 nm, typical of a folded globular protein, is observed for all species but T279‐L11. Indeed, T279‐L11 shows a 2 nm red‐shifted maximum emission peak and a 1.9‐fold increased emission intensity, suggesting a less rigid anchoring of the PLP coenzyme which gives rise to a reduced fluorescence quenching, that is, a reduced energy transfer of the intrinsic fluorescence signal to the internal aldimine (Figure 2C). On the other hand, p.L11 shows a decreased intrinsic fluorescence emission intensity, suggesting a more rigid environment that favors the energy transfer to the PLP‐Lys_209_ complex. The latter changes are not associated with an increased exposure of hydrophobic surfaces, as shown by the ANS emission fluorescence spectra (Figure S1B), thus excluding the occurrence of gross structural changes.

We then measured the kinetic parameters of the transamination reaction catalyzed by each species (Figure S2, Table 3). All variants show *k*_*cat*_ values of the same order of magnitude, indicating that p.I279T is not critical for catalysis. However, T279‐L11 displays a *K*_*m*_ value significantly higher compared with the other species, thus suggesting that the combined variants could affect the orientation of residues surrounding the active site involved in the binding of the substrate, translating into a reduced affinity for the substrate.

**TABLE 3:** Steady‐state kinetic parameters of AGT‐Ma, AGT‐Mi, AGT‐L11, and T279 mutant proteins for the alanine–glyoxylate pair.

	Substrate	Cosubstrate	*k*_*cat*_ (s^−1^)	K_m (Alanine)_ (mM)	K_m (Glyoxylate)_ (mM)	*k*_*cat*_/K_m_ (s^−1^ mM^−1^)
AGT‐Ma	L‐Alanine	Glyoxylate	45 ± 2^a^	31 ± 4^a^		1.45 ± 0.2^a^
	Glyoxylate	L‐Alanine	45 ± 3^a^		0.23 ± 0.05^a^	196 ± 44^a^
AGT‐Mi	L‐Alanine	Glyoxylate	33 ± 5^b^	28 ± 2^b^		1.2 ± 0.2^b^
	Glyoxylate	L‐Alanine	37 ± 1^b^		0.22 ± 0.01^b^	168 ± 8^b^
AGT‐L11	L‐Alanine	Glyoxylate	32 ± 1^c^	43 ± 4^c^		0.74 ± 0.07^c^
	Glyoxylate	L‐Alanine	36 ± 1^c^		0.34 ± 0.04^c^	105 ± 13^c^
T279‐Ma	L‐Alanine	Glyoxylate	34 ± 1	35 ± 4		0.94 ± 0.1
	Glyoxylate	L‐Alanine	29 ± 1		0.31 ± 0.06	96 ± 20
T279‐Mi	L‐Alanine	Glyoxylate	33 ± 1	46 ± 6		0.70 ± 0.1
	Glyoxylate	L‐Alanine	32 ± 3		0.71 ± 0.1	45 ± 7
T279‐L11	L‐Alanine	Glyoxylate	37 ± 5	114 ± 3		0.31 ± 0.04
	Glyoxylate	L‐Alanine	36 ± 1		0.22 ± 0.03	166 ± 24

^a^ From ref. [1].

^b^ From ref. [30].

^c^ From ref. [15].

Overall, the data indicate that p.I279 is not critical for the functional properties of AGT, but its mutation to Thr causes slight changes in the microenvironment of the active site, with the most prominent effects observed when combined with the p.P11L substitution.

We then examined the effects of the mutation on the general stability of the enzyme by comparing the resistance to thermal stress, the aggregation propensity, and the susceptibility to degradation of the p.I279 variant with the AGT‐Ma, AGT‐Mi, or AGT‐L11 haplotypes. The melting temperatures of each species (Table 4) show that: (i) the substitutions of the minor allele partly reduce the overall thermodynamic stability of AGT, as previously reported [8]. However, the main change is caused by p.L11, while p.M340 plays a stabilizing role, as shown by the 7° reduction of the Tm of p.L11 compared with the 4° reduction of AGT‐Mi; (ii) the p.T279 variant causes a slight reduction of thermal stability when inherited on the major or minor haplotype. However, the concomitant presence of p.T279 and p.L11 leads to a more pronounced effect, as demonstrated by the more than 20° drop in the melting temperature. None of the variants under study increased the tendency to proteolytic degradation upon prolonged incubation in the presence of a low‐specificity protease (Figure S1C). Moreover, all but T279‐L11 are stable when incubated at physiological temperature, ionic strength, and pH. Notably, T279‐L11 is the only species that displays a significant tendency to aggregation (*k*_*obs*_ = 36 s^−1^), a behavior that is not prevented by the addition of exogenous PLP (Figure 2D), thus suggesting that coenzyme binding is not involved in the process.

**TABLE 4:** Transition midpoints of thermal denaturation I of AGT‐Ma, AGT‐Mi, AGT‐L11, and T279 mutants.

	Melting temperature (°C)
AGT‐Ma	77 ± 0.2
AGT‐Mi	73 ± 0.1
AGT‐L11	70 ± 0.2
T279‐Ma	73 ± 0.2
T279‐Mi	67 ± 0.5
T279‐L11	50 ± 1.5

Together, these data indicate that the p.I279T substitution causes an AGT conformational change that reduces the overall stability of the protein and increases its propensity to aggregation only when it is coinherited with p.L11 without p.M340.

### 3.4 Cellular Effects of the p.I279T Mutation in Mammalian Cells

**FIGURE 3:**
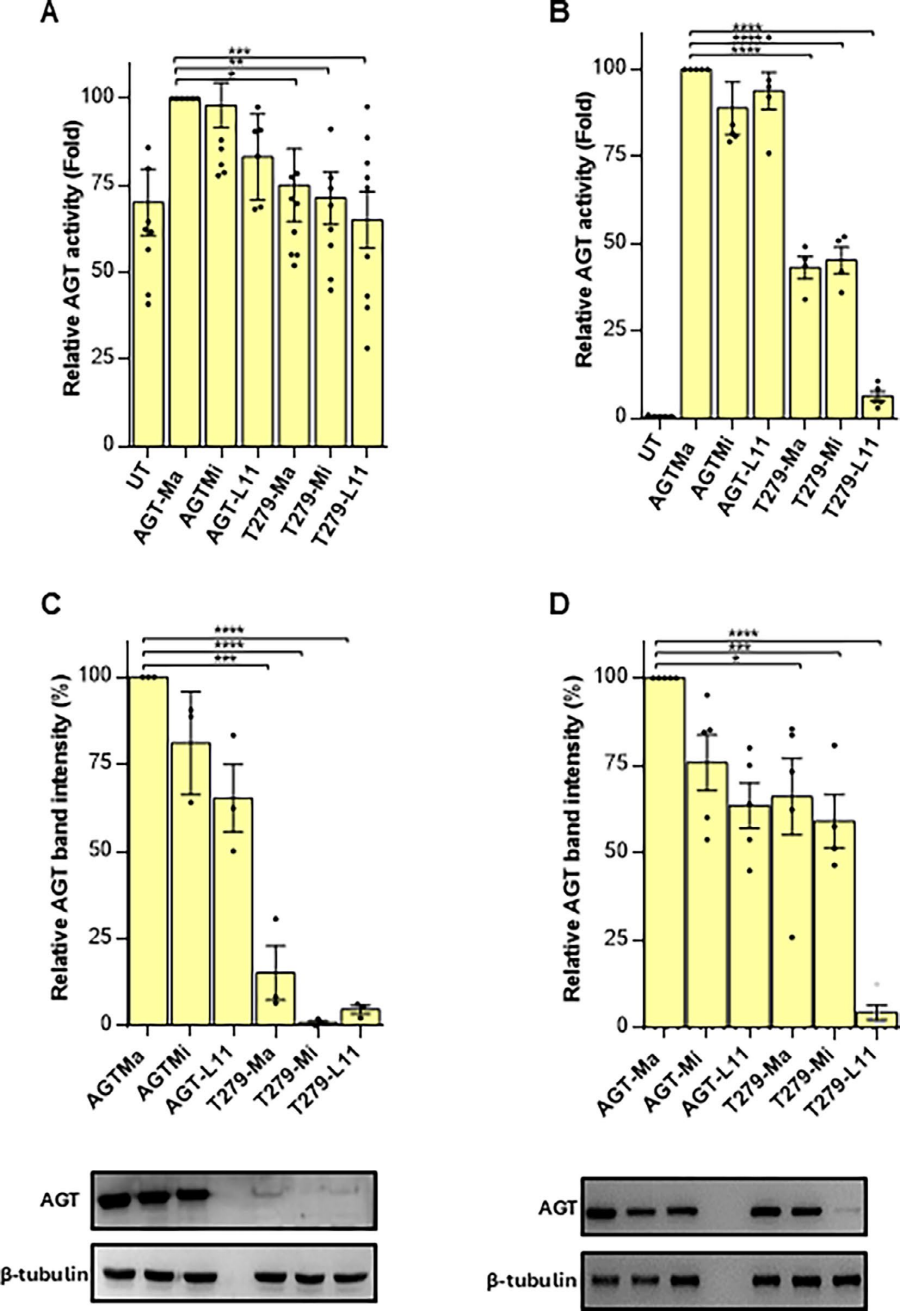
Cellular studies of AGT‐Ma, AGT‐Mi, AGT‐L11, and the p.T279 mutant proteins transiently expressed in *AGXT1*‐KO HepG2 cells (A and C) or CHO cells (B and D). Specific AGT transaminase activity and AGT protein levels were assessed in the soluble fraction of cell lysates by a spectrophotometric assay and immunoblotting, respectively. The mean value of specific AGT transaminase activity in cells expressing AGT‐Ma was assumed as 100 to facilitate the assessment of the changes. All other transaminase activity values were related to that of AGT‐Ma, whose value in HepG2 and CHO cells is 7.4 ± 0.4 and 217.7 ± 5.2 nmol of pyruvate/min/mg protein, respectively. Graphs C and D represent the densitometric analysis of AGT protein levels as the relative expression of AGT normalized to β‐tubulin levels with respect to AGT‐Ma transfected cells. Protein expression levels are not shown for untreated control cells, in both cases, as they are not significant for densitometric analysis. Representative blots out of three independent experiments are shown. Results are mean ± SEM (*n* = 3). *p < 0.05, **p < 0.01, ***p < 0.0005, ****p < 0.0001 versus AGT‐Ma.

We compared the properties of T279‐Ma, T279‐Mi, and T279‐L11 with those of AGT‐Ma, AGT‐Mi, and AGT‐L11 upon expression in mammalian cells. We first chose as a model HepG2 cells knocked out for the endogenous *AGXT* gene (*AGXT*‐KO HepG2) [29] transiently transfected with pcDNA3.1 vectors encoding the variants under study, and analyzed the AGT expression levels and transaminase‐specific activity. The data reported in Figure 3A,C show that AGT‐Mi displays slightly reduced protein levels and specific transaminase activity compared to AGT‐Ma, and that p.L11 alone leads to effects similar to those observed with the minor allele, in agreement with previous molecular data indicating that the p.P11L substitution is the main reason for structural changes leading to reduced intracellular stability of AGT‐Mi [10, 15, 17]. On the other hand, the presence of the p.T279 variant on each of the three backgrounds leads to a strong reduction in protein levels in both the soluble and the total cellular lysate, as well as to transaminase‐specific activity values that do not significantly differ from those of the untransfected control (Figure 3A,C). Although these data confirm that the conformational changes caused by p.T279 strongly impact AGT intracellular behavior, they do not provide evidence of differences based on the genetic background.

Therefore, we expressed the variants in CHO cells, which do not exactly mimic the physiological AGT levels and environment in human liver, but can allow maximized expression to possibly appreciate differences among the AGT haplotypes with p.T279. The transient transfection of AGT‐Ma, AGT‐Mi, and the p.L11 variant in CHO cells gives rise to similar AGT levels in the soluble fraction of the lysate, although the specific activity of AGT‐Mi and AGT‐L11 is reduced by approximately 30% as compared with AGT‐Ma (Figure 3B,D), in agreement with previous molecular studies indicating that the minor allele slightly reduces AGT intracellular stability, mainly due to p.L11. As compared with AGT‐Ma and AGT‐Mi, T279‐Ma and T279‐Mi show ~60%‐reduced transaminase‐specific activity and ~ 40%‐reduced protein levels. Furthermore, T279‐L11 displays just 10% residual transaminase‐specific activity and 7% protein levels as compared with AGT‐Ma, thus indicating that the concomitant presence of the two variants, in the absence of p.M340, could give rise to a synergic impairment of the folding/stability of AGT.

### 3.5 Molecular Modeling of p.I279T

**FIGURE 4:**
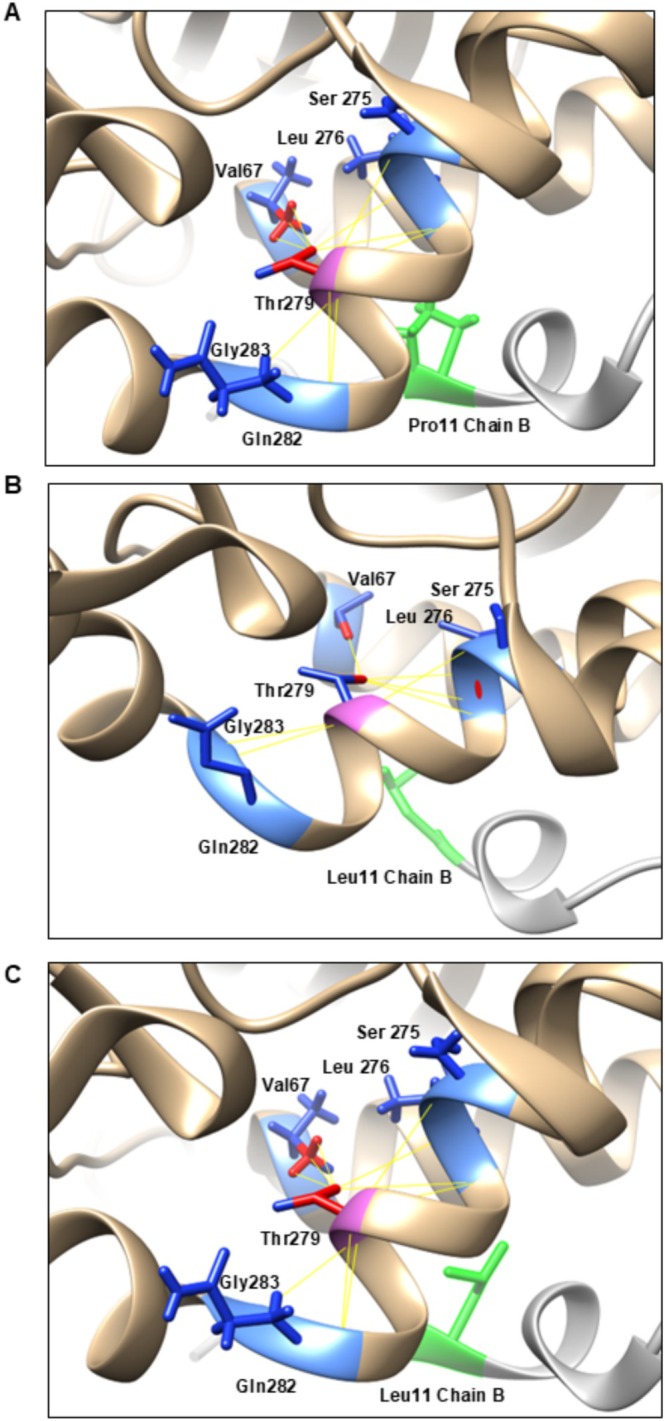
In silico analysis of the predicted microenvironment of a Thr residue at position 279 on AGT‐Ma, AGT‐Mi, and AGT‐Ma in the presence of the p.P11L substitution. Cartoon representation of the predicted 3D structure of AGT mutants (A) T279‐Ma, (B) T279‐Mi, and (C) T279‐L11. Chain A, tan; Chain B, white. Residues belonging to helix 265–283 or interacting with p.T279 around a 5 Å sphere are shown as sticks and colored as follows: T279 as purple sticks, P11 or L11 (Chain B) as green sticks, and residues involved in the interaction as cornflower blue sticks. Interactions are highlighted as yellow lines. Structure analysis of clashes is highlighted in red. In silico models were obtained using UCSF Chimera (Alpha version 1.17) from the available crystal structure of AGT‐Ma (PDB ID: 5F9S) and AGT‐Mi (PDB ID: 7NS7).

We performed an in silico mutagenesis study to gain insights into the possible structural bases of the synergism between p.T279 and p.L11. p.I279 is located on α‐helix 265–283 and is surrounded by nonpolar amino acids that define a hydrophobic region. The p.I279T substitution introduces a polar residue that alters the hydrophobic microenvironment, probably causing a destabilization of this region, without significantly affecting the active site that is located at a distance > 10 Å. However, the N‐terminal arm of the neighboring subunit containing p.P11 is located in front of the α‐helix 265–283 at a distance of 7 Å from p.I279 (Figure 4). Thus, the substitution p.P11L can increase the fluctuation of the N‐terminal tail, exacerbating the effects of p.I279T. In addition, structural and in vitro data indicate that the p.I340M substitution exerts a stabilizing effect by reducing the overall dynamics of AGT, especially in the region comprising p.P11 [17]. Based on this observation, we can hypothesize that the conformational alterations caused by p.T279 in the presence of p.L11 could be more pronounced in the absence of the stabilizing effect of p.M340.

## 4 Discussion

In recent years, remarkable advances in sequencing technologies have revolutionized diagnosing rare diseases, making it relatively easy to identify pathogenic variants within target genes in a proband. Sequence data from large populations of normal individuals, such as gnomAD, has also aided differentiation of likely pathogenic from benign changes (monogenic disease alleles are rare). However, in some cases, genetic information is not so clear as to fully prove a diagnosis, in particular when the clinical significance of a newly identified variant is unclear or there is contradictory data (such as the variant being too common to be a monogenic disease allele). In these cases, functional studies can be critical to support the pathogenicity of variants and provide a definitive diagnosis [31, 32]. In PH1, this aspect is further complicated by the presence in *AGXT* of two haplotypes consisting of p.P11L and p.I340M, which can modulate the phenotypic expression of other missense changes, potentially affecting their pathogenicity [12, 33, 34].

In this work, we studied the molecular and cellular effects of the p.I279T variant. At the clinical level, *AGXT* p.I279T has normally been dismissed as benign because its population frequency is higher than expected for a pathogenic change, and experimental data suggest that AGT function is not altered when this substitution occurs on the major allele, where it is normally found [19, 20]. Nevertheless, *AGXT* p.I279T has been occasionally found on the minor allele, and family data suggest it to be pathogenic, thus implying that the *AGXT* haplotype could be critical to determining pathogenicity [20]. Here we strengthen the genetic data with 6 families and 8 individuals with a clear PH phenotype, either homozygous for *AGXT* p.I279T or that have this variant biallelic with a known PH1 pathogenic change. Interestingly, we describe genetic details of the likely pathogenic haplotype, with presumed recombination between p.P11L and p.I340M, resulting in the p.L11‐T279‐I340 haplotype proven or inferred in each case. Given the distance between these variants and the strong linkage disequilibrium, it is possible that a single historic crossover gave rise to the rare p.L11‐T279‐I340 haplotype that we propose is uniquely pathogenic.

Data collected on purified AGT indicate that the p.I279T substitution does not cause a gross structural alteration and is not critical for the transamination reaction, but it affects the microenvironment of the active site when present concomitantly with p.L11 in the absence of p.M340, as shown by circular dichroism, fluorescence, and kinetic data [19]. However, p.L11 and p.T279 synergistically reduce AGT thermodynamic stability and increase its aggregation propensity, without altering susceptibility to proteolytic cleavage. The AGT destabilization caused by the p.I279T substitution is confirmed by cellular studies. Indeed, when expressed in a PH1 model made up of HepG2 cells, all AGT haplotypes bearing p.T279 show a significant reduction in specific transaminase activity caused by the presence of strongly reduced protein levels. Interestingly, when p.T279 on various haplotypes is characterized in CHO cells (to maximize expression), the synergistic effect of the concomitant combination of p.L11 and p.T279 in the absence of the stabilizing effect of p.M340 becomes evident from the higher reduction in specific activity and protein levels of T279‐L11 as compared with T279‐Ma and T279‐Mi.

The structural reasons underlying the T279‐L11 synergism are likely due to conformational changes of the N‐terminus caused by both the p.P11L substitution, which increases the flexibility of the arm as already established from structural and molecular studies [9, 17, 35], and the p.I279T change, as suggested by our molecular modeling studies. An analogous functional synergism between polymorphic and pathogenic changes has been previously observed in PH1, e.g., for the p.G170R substitution, which is pathogenic on the minor allele [9], or the p.Gly41Arg (p.G41R) and p.Ile56Asn (p.I56N) changes, whose effects are worsened by the concomitant presence of the minor haplotype [12, 14, 33, 36]. However, differently from what has been observed for p.G170R (and p.G41R and p.I56N), in the case of p.I279T the phenotypic expression of the synergism depends not only on Leu at position 11, but also requires Ile at residue 340. This is indicated by our finding that (i) the p.T279 variant has only been described in PH1 patients on the p.L11‐I340 background, and (ii) the molecular and cellular effects of the p.I279T substitution are significantly more evident with the p.L11 allele alone than on the minor allele. This effect is because the p.M340 variant plays a stabilizing role in AGT. Indeed, crystallographic and molecular dynamics studies have suggested that the Ile340‐to‐Met substitution could partly counterbalance the increased flexibility of the N‐terminus caused by Pro11‐to‐Leu [6, 15, 17].

From the limited clinical data, it appears that the phenotypic severity associated with p.I279T is quite variable, ranging from kidney failure at 2 years when found with a truncating variant to no kidney failure at 56 years when in homozygosity. Pyridoxine treatment has been shown to be effective for some PH1 pathogenic changes (notably p.G170R, found on the minor allele) by aiding folding and trafficking, and it remains to be seen whether this treatment will be effective for p.T279 (on the p.L11‐I340 haplotype).

Overall, our results expand the understanding of the molecular pathogenesis of PH1 by highlighting that the pathogenicity of a variant not only depends on the Ma‐ or Mi haplotype but can also be differently tuned by the two variants making up those haplotypes. It follows that a correct diagnosis of the disease, in particular in patients with the p.I279T variant, requires a complete analysis of the genetic testing results that includes the two haplotype variants, as well as family studies to determine the causal relationship between genotype and phenotype. In addition, it highlights possible modifiers of the PH1 phenotype. This includes, as previously suggested if an in‐frame pathogenic variant is found on the major or minor allele [12, 33]. But also rare cases where the pathogenic change is found on the split, p.L11‐p.I340 haplotype, and when p.I279T on the major allele (0.29% of alleles) is found in cis with a pathogenic allele. The establishment of more complete genotyping may be of prognostic value and will be crucial to implementing personalized medicine approaches for PH1.

## Author Contributions

L.R.: conceptualization, methodology, formal analysis, investigation, writing original draft, writing – review and editing. A.G.C.: performed the genetic studies, writing – review and editing. G.P.: investigation, formal analysis. D.J.S.: reviewed the clinical data, writing – review and editing. J.C.L.: extracted the clinical data, writing – review and editing. G.R.: performed genetic studies, extracted the clinical data, writing – review and editing. B.C.: conceptualization, supervision, project administration, writing – original draft, writing – review and editing, funding acquisition. P.C.H.: conceptualization, supervision of the genetic studies, writing and editing the original and final drafts, funding acquisition.

## Conflicts of Interest

B.C. has received research funding from Novo Nordisk Pharmaceuticals. P.C.H.: none related to this study, but has performed research studies for: Espervita, Navitor, Acceleron, Jemincare, and Regulus; obtained licensing fees from Bayer, Sanofi, Vertex, Mitobridge, Maze Therapeutics, Calico Life Sciences; and consulted for Vertex, Mitobridge, Regulus, Otsuka, Janssen, Maze Therapeutics, and RenasantBio. D.J.S. has received research funding from Alnylam and Novo Nordisk, and consulting funding from Arbor and Advicenne. G.R.: consultant for Novo Nordisk.

## Supporting information


Data S1.

## Data Availability

The data that support the findings of this study are available from the corresponding author upon reasonable request.
