# Supplementary material for: A Minor Haplotype Variant Determines the Pathogenicity of the p.Ile279Thr Substitution in the Primary Hyperoxaluria Type 1 Gene, AGXT
Source: J Inherit Metab Dis. 2025 Jun 11;48(4):e70052. doi: 10.1002/jimd.70052 (PMC12152710; doi:10.1002/jimd.70052)
Supplement: Supplementary file 1 — Data S1. [file JIMD-48-0-s001.docx]

**Supplementary Data**

**Supplementary Table 1.** **Prediction of the effects of the p.I279T substitution on the structural dynamics of AGT**. *In silico* prediction of the effects of the single-point sustitution p.I279T on AGT folding and flexibility as shown by ΔΔS ENCoM and ΔΔG values obtained from freely available webservers, DunaMut, DDGun Prediction, DDMut, MutPred2.^1–3^ Analyses were performed using the available crystal structure of AGT-Ma (PDB code: 5F9S) or AGT-Mi (PDB code: 7NS7).

| **Tool** | **Output data** | **T279-Ma** | **T279-Mi** | **T279-L11** |
| --- | --- | --- | --- | --- |
| **DynaMut** | **∆∆G (Kcal/mol)** | -2.277  Destabilizing | 0.765  Stabilizing | -2.277  Destabilizing |
|  | **∆∆S_Vib_ ENCoM**  **Kcal.mol^-1^.K^-1^** | 0.316  Increase of molecule flexibility | -0.537  Decrease of molecule flexibility | 0.316  Increase of molecule flexibility |
|  | **∆∆S ENCoM**  **Kcal/mol**  **NMA Based Prediction** | -0.253  Destabilizing | 0.430  Destabilizing | -0.253  Destabilizing |
|  | **∆∆G mCSM**  **Kcal/mol**  **Structure-Based Predictions** | -2.367  Destabilizing | -2.250  Destabilizing | -2.367  Destabilizing |
|  | **∆∆G SDM**  **Kcal/mol** | -3.460  Destabilizing | -2.690  Destabilizing | -3.460  Destabilizing |
|  | **∆∆G DUET**  **Kcal/mol** | -2.702  Destabilizing | -2.472  Destabilizing | -2.702  Destabilizing |
| **DDGun Prediction** | **∆∆G [3D]/Stability** | -1.6  Decrease | -1.8  Decrease | n.a |
| **DDMut** | **∆∆G^Stability wt->mt^**  **(Kcal/mol)** | 0.02  Stabilizing | 0.04  Stabilizing | 0.02  Stabilizing |
| **MutPred2** | **MutPred2 score** | 0.792 | 0.833 | 0.821 |

**Supplementary Fig.1. Spectral and stability studies on purified mutant proteins (A)** Far-UV CD spectra of the indicated species in the presence of 20 μM exogenous PLP **(B)** Fluorescence emission spectra of the indicated species in the presence of 20 µM PLP incubated with 1 mM ANS at 25 °C for 1 h. Excitation was set at 365 nm. Protein concentration was kept equal to 1 µM in 100 mM potassium phosphate buffer, pH 7.4. **(C)** Limited proteolysis assays using proteinase K (100/1 protein/proteinase K ratio). At indicated times, aliquots were removed, denatured in sample buffer, boiled for 5 minutes, and subjected to 12% SDS-PAGE.


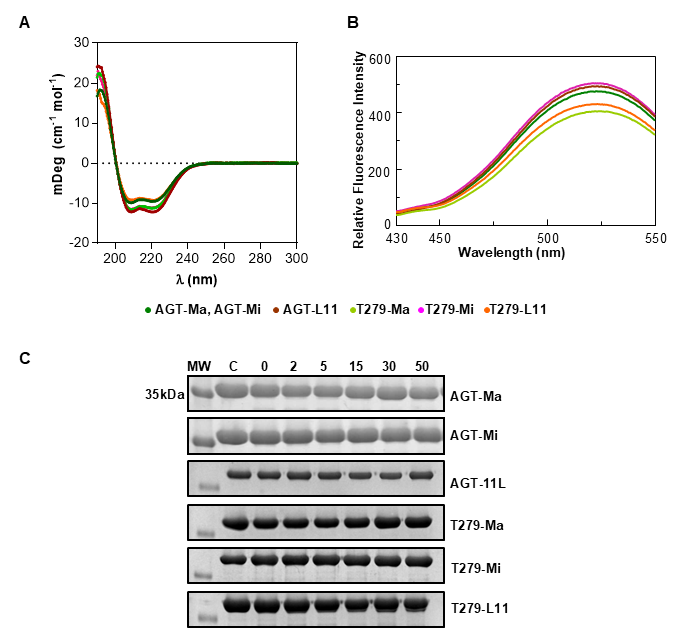


**Supplementary Fig. 2.** **Kinetic parameters for the transamination reaction of AGT-Ma, AGT-Mi, AGT-L11 and for AGT-p.I279T variants**. The graphs show the enzyme activity measured by varying the substrate concentrations (L-alanine in panel A, sodium glyoxylate in panel B) at a fixed saturating co-substrate concentration (10 mM sodium glyoxylate in panel A, 500 mM L-alanine in panel B). The curves represent the fitting of the data to the Michaelis-Menten equation.

**
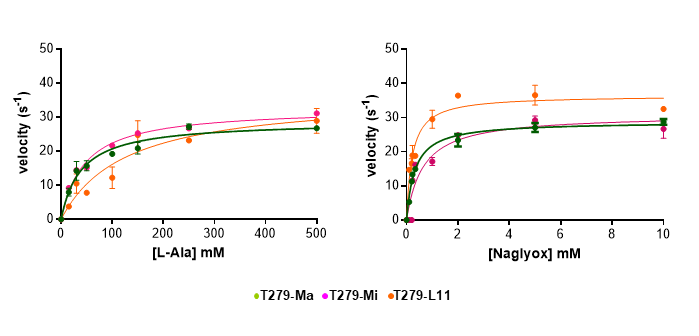
**

**B**

**A**

**Supplementary Fig. 3.** **Protein levels of Un-Treated control (UT) and AGT-Ma transiently expressed in AGXT1-KO HepG2 cells (A) or CHO cells** (**B**) Densitometric analysis of Un-Treated control (UT) and AGT-Ma protein levels assessed in the soluble fraction of cell lysates by a spectrophotometric assay and immunoblotting, respectively.


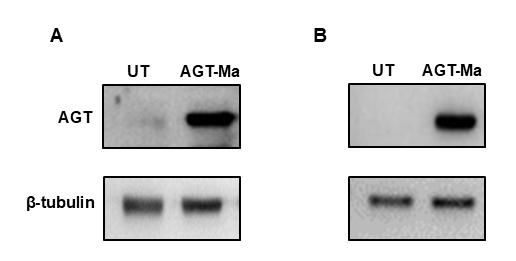


**References**

1. Rodrigues, C. H., Pires, D. E. & Ascher, D. B. DynaMut: predicting the impact of mutations on protein conformation, flexibility and stability. *Nucleic Acids Res* **46**, W350–W355 (2018).

2. Pan, Q., Nguyen, T. B., Ascher, D. B. & Pires, D. E. V. Systematic evaluation of computational tools to predict the effects of mutations on protein stability in the absence of experimental structures. *Brief Bioinform* **23**, (2022).

3. Montanucci, L. *et al.* DDGun: an untrained predictor of protein stability changes upon amino acid variants. *Nucleic Acids Res* **50**, W222–W227 (2022).
